# Divergent signaling pathways cooperatively regulate TGFβ induction of cysteine-rich protein 2 in vascular smooth muscle cells

**DOI:** 10.1186/1478-811X-12-22

**Published:** 2014-03-28

**Authors:** Meng-Ling Wu, Chung-Huang Chen, Yung-Tsang Lin, Yuan-Jyun Jheng, Yen-Chun Ho, Liang-Tung Yang, Linyi Chen, Matthew D Layne, Shaw-Fang Yet

**Affiliations:** 1 Institute of Cellular and System Medicine, National Health Research Institutes, Zhunan, Taiwan; 2 Institute of Molecular Medicine, National Tsing Hua University, Hsinchu, Taiwan; 3 Department of Medical Science and Institute of Bioinformatics and Structural Biology, National Tsing Hua University, Hsinchu, Taiwan; 4 Graduate Institute of Life Sciences, National Defense Medical Center, Taipei, Taiwan; 5 Department of Biochemistry, Boston University School of Medicine, Boston, Massachusetts, USA; 6 Metabolomic Research Center, China Medical University Hospital, Taichung, Taiwan; 7 Graduate Institute of Basic Medical Science, China Medical University, Taichung, Taiwan

**Keywords:** Cysteine-rich protein 2, Vascular smooth muscle cells, TGFβ, ATF2, Smad2/3

## Abstract

**Background:**

Vascular smooth muscle cells (VSMCs) of the arterial wall play a critical role in the development of occlusive vascular diseases. Cysteine-rich protein 2 (CRP2) is a VSMC-expressed LIM-only protein, which functionally limits VSMC migration and protects against pathological vascular remodeling. The multifunctional cytokine TGFβ has been implicated to play a role in the pathogenesis of atherosclerosis through numerous downstream signaling pathways. We showed previously that TGFβ upregulates CRP2 expression; however, the detailed signaling mechanisms remain unclear.

**Results:**

TGFβ treatment of VSMCs activated both Smad2/3 and ATF2 phosphorylation. Individually knocking down Smad2/3 or ATF2 pathways with siRNA impaired the TGFβ induction of CRP2, indicating that both contribute to CRP2 expression. Inhibiting TβRI kinase activity by SB431542 or TβRI knockdown abolished Smad2/3 phosphorylation but did not alter ATF2 phosphorylation, indicating while Smad2/3 phosphorylation was TβRI-dependent ATF2 phosphorylation was independent of TβRI. Inhibiting Src kinase activity by SU6656 suppressed TGFβ-induced RhoA and ATF2 activation but not Smad2 phosphorylation. Blocking ROCK activity, the major downstream target of RhoA, abolished ATF2 phosphorylation and CRP2 induction but not Smad2 phosphorylation. Furthermore, JNK inhibition with SP600125 reduced TGFβ-induced ATF2 (but not Smad2) phosphorylation and CRP2 protein expression while ROCK inhibition blocked JNK activation. These results indicate that downstream of TβRII, Src family kinase-RhoA-ROCK-JNK signaling pathway mediates TβRI-independent ATF2 activation. Promoter analysis revealed that the TGFβ induction of CRP2 was mediated through the CRE and SBE promoter elements that were located in close proximity.

**Conclusions:**

Our results demonstrate that two signaling pathways downstream of TGFβ converge on the CRE and SBE sites of the *Csrp2* promoter to cooperatively control CRP2 induction in VSMCs, which represents a previously unrecognized mechanism of VSMC gene induction by TGFβ.

## Background

Vascular smooth muscle cells (VSMCs) in the tunica media of the arteries play important roles in regulating blood pressure and vascular tone. In normal vessels, these VSMCs exhibit a quiescent and differentiated phenotype and express proteins involved in the contractile functions such as smooth muscle (SM) myosin heavy chain and SM α-actin. However, in contrast to striated muscle cells, adult VSMCs retain significant plasticity, known as phenotypic modulation. In response to arterial injury, VSMCs de-differentiate, downregulate SM marker genes, and change to a proliferative and migratory phenotype, leading to lesion formation and occlusive vascular disease [1]. Thus, preventing this de-differentiation might be a potential therapeutic strategy for treating vascular disease.

Cysteine-rich protein (CRP) 2, a LIM-only CRP family member [2], is highly expressed in VSMCs [3,4]. Importantly, balloon or wire artery injury reduces CRP2 expression [3,4], suggesting a critical role for CRP2 in the response to vascular injury. By gene deletion experiments, we demonstrated that a lack of CRP2 enhanced VSMC migration into the intima and increased neointima formation following arterial injury [4]. Our recent study determined that CRP2 sequesters the scaffold protein p130Cas at focal adhesions, controls lamellipodia formation and reduces cell motility. These CRP2-p130Cas complexes function to blunt VSMC migration [5]. Therefore, maintaining or upregulating CRP2 expression during vascular injury might serve as a protective mechanism against intimal thickening.

The multifunctional cytokine TGFβ contributes to the pathogenesis of atherosclerosis and restenosis [1,6]. During progressive intimal thickening following balloon angioplasty, neointimal SMCs produce TGFβ and it acts as a growth regulatory factor [6]. The importance of this autocrine TGFβ pathway in vascular disease was established through studies selectively inhibiting TGFβ mRNA in a rat vascular injury model in vivo, which resulted in blunted neointimal formation [7]. Some of the effects of TGFβ in VSMCs are controversial. For example, serum concentrations of active TGFβ are significantly reduced in patients with advanced atherosclerosis, suggesting TGFβ is a key inhibitor of atherosclerosis [8]. TGFβ stimulates SMC differentiation marker gene expression [1,9,10] and inhibition of TGFβ signaling using neutralizing anti-TGFβ antibodies accelerates the development of atherosclerotic lesions in apoE-deficient mice [11]. Further supporting a protective role of TGFβ against vascular lesion formation, targeted disruption of TGFβ-Smad3 signaling enhances neointimal hyperplasia after arterial injury [12].

We identified in a previous study that TGFβ induces CRP2 expression in VSMCs and TGFβ treatment reduces wild type but not *Csrp2* (mouse CRP2 gene symbol)-deficient VSMC migration, demonstrating the functional importance of CRP2 induction by TGFβ in regulating VSMC migration [13]. TGFβ upregulates CRP2 expression via a CRE promoter element and transcription factor ATF2 [13]; however, the detailed signaling mechanisms underlying TGFβ induction of CRP2 remain unclear. The goal of the present study was to delineate the signaling pathways by which TGFβ upregulates CRP2 expression, which might provide an opportunity for developing targeted strategies to reduce intimal thickening.

## Results

### TGFβ induces CRP2 expression through Smad2/3 and ATF2

**Figure 1:**
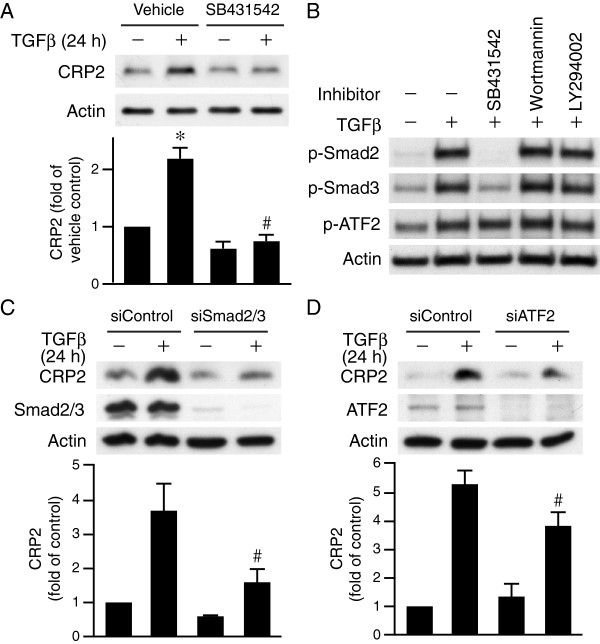
**TGFβ induces CRP2 expression through Smad2/3 and ATF2. (A)** TβRI kinase activity contributes to CRP2 induction. VSMCs were pretreated with vehicle or TβRI kinase inhibitor SB431542 (10 μM) for 30 min before stimulation with TGFβ (10 ng/ml) for 24 h. Total proteins were then harvested for Western blot analysis to detect CRP2 expression. SB431542 significantly decreased TGFβ-induced CRP2 expression. Values are mean ± S.E. of at least three experiments. *P < 0.05 vs. control (− TGFβ); ^#^*P* < 0.05 vs. TGFβ-stimulated vehicle group. **(B)** TβRI kinase activity is required for activation of Smad2/3 but not ATF2. VSMCs were pretreated with vehicle, SB431542, PI3K inhibitor Wortmannin (1 μM), or LY294002 (10 μM) for 30 min prior to stimulation with or without TGFβ for 15 min. Activation of Smad2/3 and ATF2 was then determined with Western blot analysis. **(C-D)** VSMCs were transfected with 20 nM control siRNA, Smad2/3 siRNA **(C)** or ATF2 siRNA **(D)** using Lipofectamine RNAiMAX transfection reagent and then stimulated with or without TGFβ for 24 h. Western blot analysis was performed to detect CRP2, Smad2/3, or ATF2 levels. The membranes were subsequently probed with actin for loading control. Values are mean ± S.E. of at least three experiments. ^#^*P* < 0.05 vs. TGFβ-stimulated siControl group.

To investigate the signaling pathways that mediate CRP2 induction by TGFβ, we first examined type I TGFβ receptor (TβRI) downstream signaling. We pretreated VSMCs with vehicle or TβRI kinase inhibitor SB431542 for 30 min, followed by stimulation with or without TGFβ for 24 h and then examined CRP2 expression levels. SB431542 significantly reduced TGFβ-induced CRP2 expression (Figure 1A), indicating TβRI kinase activity is required for TGFβ induction of CRP2. It is well established that Smad2/3 transmits TGFβ signaling [14], thus we examined Smad2/3 activation. Indeed, TGFβ increased phosphorylation levels of Smad2 and Smad3 in VSMCs (Figure 1B). In addition, as previously reported [13], TGFβ also increased ATF2 phosphorylation (Figure 1B). Interestingly, SB431542 blocked TGFβ-induced activation of Smad2 and Smad3 but did not block ATF2 phosphorylation (Figure 1B). PI3K has also been implicated in TGFβ signaling [15], thus we determined whether PI3K pathways participate in this regulation by treating cells with PI3K inhibitors. Wortmannin or LY294002 did not affect ATF2 or Smad2/3 phosphorylation (Figure 1B). These results suggest that in VSMCs, TβRI mediates TGFβ activation of Smad2/3 whereas neither TβRI kinase activity nor PI3K signaling is involved in TGFβ dependent stimulation of ATF2. To define the role of Smad2/3 and ATF2 in CRP2 upregulation, we used siRNA to suppress their expression. In comparison with control siRNA, knockdown of Smad2/3 or ATF2 abrogated TGFβ-induced CRP2 expression (Figure 1C-D), supporting the concept that both Smad2/3 and ATF2 contribute to CRP2 induction.

### ATF2 activation by TGFβ is independent of TAK1 and TRAF6

**Figure 2:**
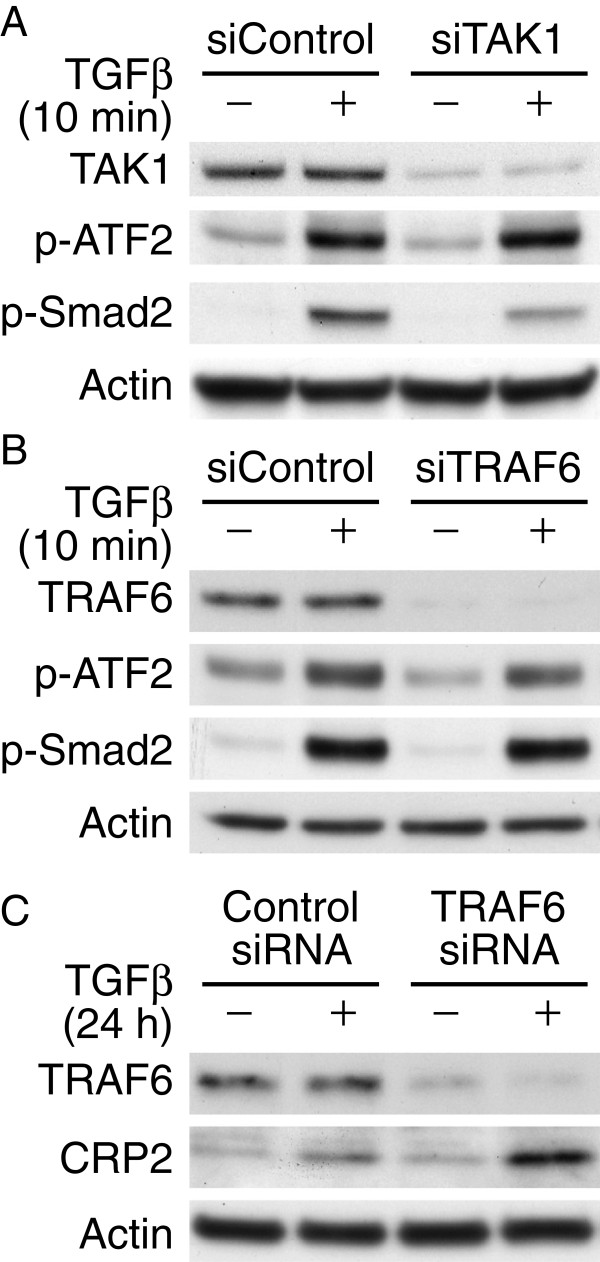
**Neither TAK1 nor TRAF6 mediates TGFβ-induced ATF2 activation.** VSMCs were transfected with 20 nM TAK1 siRNA **(A)** or TRAF6 siRNA **(B)** using Lipofectamine RNAiMAX transfection reagent and then stimulated with or without TGFβ for 10 min. Control siRNA was also transfected as a control. Western blot analysis was performed to detect TAK1, TRAF6, and phosphorylation of ATF2 and Smad2. The membranes were subsequently probed with actin for loading control. **(C)** VSMCs were transfected with control or TRAF6 siRNA and then treated with or without TGFβ for 24 h. Total proteins were then isolated for Western blot analysis to detect TRAF6 and CRP2 expression. The membranes were subsequently probed with actin for loading control. Representative blots of at least three independent experiments are shown.

It has been shown in epithelial cells and fibroblasts that independent of TβRI kinase activity, TGFβ activates TAK1 signaling through interaction of TβRI with TRAF6, whereas Smad2 activation is not dependent on TRAF6 [16,17]. Thus, we examined whether TGFβ activates ATF2 through TAK1 and TRAF6 in VSMCs. We transfected VSMCs with siRNA to TAK1 or TRAF6, or control siRNA and then stimulated cells with or without TGFβ for 10 min. Western blot analysis showed that knocking down TAK1 did not alter ATF2 activation although it partially reduced Smad2 phosphorylation (Figure 2A), indicating TAK1 was not involved in TGFβ-induced ATF2 phosphorylation in VSMCs. TRAF6 knockdown did not affect TGFβ-induced ATF2 or Smad2 phosphorylation (Figure 2B). Moreover, knocking down TRAF6 did not abrogate TGFβ induction of CRP2 protein expression after 24 h (Figure 2C).

### TβRI kinase-independent ATF2 activation by TGFβ

**Figure 3:**
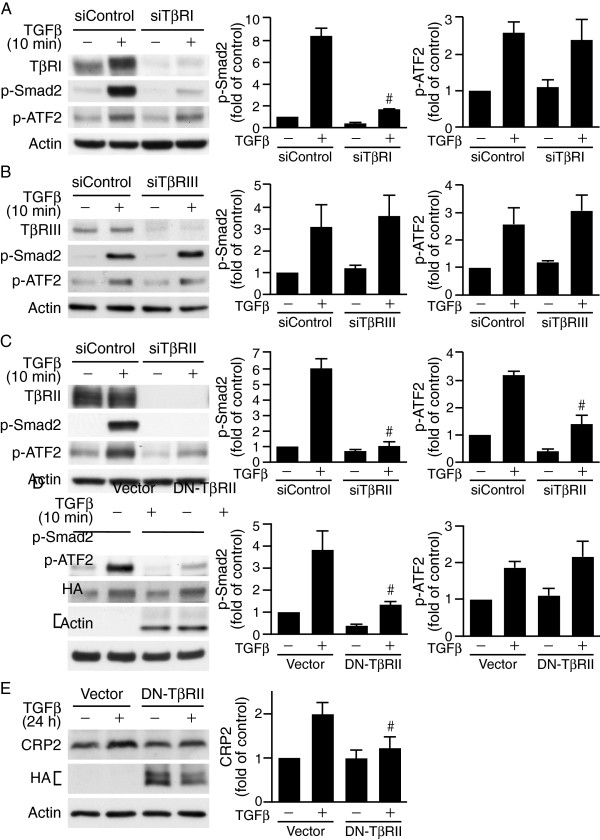
**Type II TGFβ receptor is crucial in mediating TGFβ-induced ATF2 signaling. (A-C)** VSMCs were transfected with 20 nM control siRNA or siRNA to different types of TGFβ receptors. Following 24 h recovery in growth media and 24 h serum-starvation, cells were stimulated with or without TGFβ for 10 min. Western blot analysis was performed to detect different TGFβ receptor expression levels and phosphorylation of Smad2 and ATF2. **(A)** TβRI knockdown inhibits Smad2 phosphorylation but not that of ATF2. ^#^*P* < 0.05 vs. TGFβ-stimulated siControl group. **(B)** TβRIII knockdown does not affect TGFβ activation of ATF2 or Smad2. **(C)** TβRII knockdown attenuates TGFβ activation of ATF2 and Smad2. ^#^*P* < 0.05 vs. TGFβ-stimulated siControl group. **(D)** Dominant-negative TβRII (DN-TβRII) impairs Smad2 but not ATF2 activation. VSMCs were electroporated with control vector or HA-tagged DN-TβRII (HA-TβRII(∆Cyt)) and then stimulated with or without TGFβ for 10 min. Western blot analysis was performed to assess phosphorylation of Smad2 and ATF2. Overexpression of DN-TβRII was evaluated by probing Western blots with HA antibody. ^#^*P* < 0.05 vs. TGFβ-stimulated vector control group. **(E)** DN-TβRII impairs TGFβ-induced CRP2 induction. VSMCs were electroporated with control vector or HA-TβRII(∆Cyt) and stimulated with TGFβ for 24 h. Total proteins were then isolated for Western blot analysis to detect CRP2 protein levels. The blots were then probed with HA antibody to detect HA-TβRII(∆Cyt) expression. ^#^*P* < 0.05 vs. TGFβ-stimulated vector control group. The membranes were subsequently probed with actin for loading control. Representative blots of at least three independent experiments are shown.

Upon ligand binding, TβRII recruits TβRI into an active heterotetrameric signaling complex and transphosphorylates TβRI to activate its kinase function [18]. TβRI can then phosphorylate Smad2/3 for TGFβ signaling. Interestingly, we found that inhibiting TβRI kinase activity blocked TGFβ-induced Smad2/3 but not ATF2 activation (Figure 1B). This ATF2 phosphorylation is not mediated through TRAF6 either (Figure 2). To confirm further that ATF2 phosphorylation is independent of TβRI, we knocked down TβRI expression using siRNA. Silencing of TβRI abrogated TGFβ-induced Smad2 phosphorylation but not ATF2 phosphorylation (Figure 3A), suggesting indeed TβRI is not required for TGFβ-induced ATF2 activation.

Type III TGFβ receptor (TβRIII) has been demonstrated to bind and present TGFβ to type II TGFβ receptor (TβRII), thereby increases type II receptor binding affinity and cell responsiveness to TGFβ [19]. To determine whether TβRIII participates in the ATF2 activation, we knocked down TβRIII levels in VSMCs by TβRIII siRNA, cells were then stimulated with or without TGFβ. Compared with control siRNA, knocking down TβRIII levels did not affect the activation of ATF2 or Smad2 at the 10 min early time point (Figure 3B) or affecting CRP2 protein expression 24 h later (data not shown). These results suggest that TβRIII did not affect TGFβ signaling in the CRP2 induction. We then knocked down TβRII expression by siRNA and examined phosphorylation of these signaling molecules. Silencing TβRII not only reduced ATF2 activation but also that of Smad2 (Figure 3C), indicating the critical importance of TβRII in mediating TGFβ signaling. To further assess the role of TβRII, we transfected VSMCs with a dominant-negative type II receptor HA-TβRII(∆Cyt) [20], which has an HA tag at the N-terminus but lacks C-terminal kinase domain and is thus incapable of phosphorylating and activating the type I receptor. Interestingly, TβRII(∆Cyt) attenuated Smad2 phosphorylation as expected but not the activation of ATF2 (Figure 3D). In addition, TβRII(∆Cyt) attenuated TGFβ-induced CRP2 expression 24 h later (Figure 3E).

### Src family kinase mediates TβRII-dependent TGFβ activation of RhoA-ROCK and ATF2 in VSMCs

**Figure 4:**
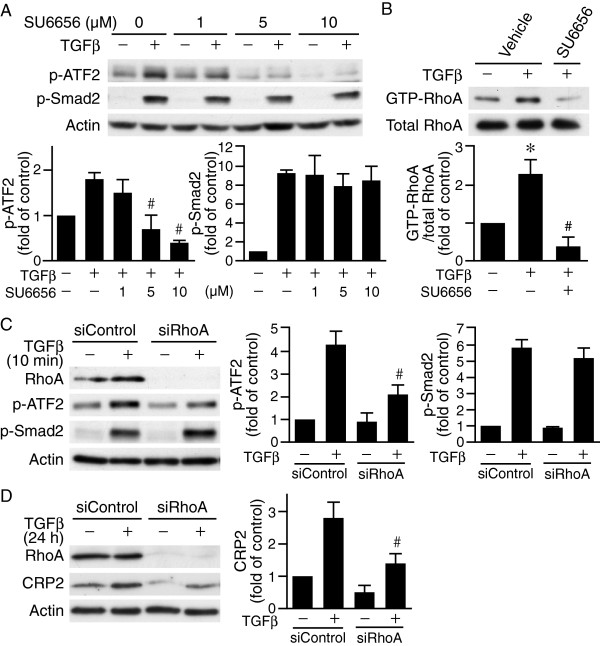
**Src family kinase mediates TβRII-dependent TGFβ activation of RhoA and ATF2 in VSMCs. (A)** Src family kinase inhibitor SU6656 dose-dependently reduces TGFβ activation of ATF2 but not that of Smad2. VSMCs were pretreated with increasing concentrations of SU6656 for 30 min and then stimulated with TGFβ for 10 min. Phosphorylation of ATF2 and Smad2 was determined by Western blot analysis. The membranes were subsequently probed with actin for loading control. ^#^*P* < 0.05 vs. TGFβ-stimulated but without SU6656 treatment group. **(B)** SU6656 abolishes TGFβ-induced RhoA activation. VSMCs were pretreated with 5 μM SU6656 for 30 min prior to stimulation with TGFβ for 10 min. RhoA activation was then determined by GST-Rhotekin-RBD assays. GTP-RhoA was subsequently eluted and subjected to Western blot analysis with an anti-RhoA antibody. To verify equal loading, 15 μg of cell lysates were run on separate gels and blots probed with an anti-RhoA antibody for total RhoA. *P < 0.05 vs. control (− TGFβ and – SU6656); ^#^*P* < 0.05 vs. TGFβ-stimulated vehicle group. **(C)** RhoA knockdown reduces TGFβ activation of ATF2 but not Smad2. VSMCs were transfected with 20 nM control siRNA or RhoA siRNA and then stimulated with or without TGFβ for 10 min. Western blot analysis was performed to detect RhoA and phosphorylation of ATF2 and Smad2. ^#^*P* < 0.05 vs. TGFβ-stimulated siControl group. **(D)** RhoA knockdown reduces TGFβ-induced CRP2 expression. VSMCs were transfected with 20 nM control siRNA or RhoA siRNA and then stimulated with or without TGFβ for 24 h. Western analysis was performed to detect RhoA and CRP2 protein levels. The membranes were subsequently probed with actin for loading control. ^#^*P* < 0.05 vs. TGFβ-stimulated siControl group. Representative blots of at least three independent experiments are shown.

We next investigated how TβRII mediates ATF2 activation independent of TβRI in VSMCs. In JEG3 choriocarcinoma cells, Src kinase is involved in TGFβ-induced early signaling [21]. In addition, c-Src has been reported to mediate TGFβ-induced SMC gene expression [22]. We therefore examined whether in VSMCs Src activation was required to transmit TGFβ signaling for ATF2 activation. The selective Src family kinase inhibitor SU6656 dose dependently abrogated TGFβ-induced ATF2 activation (Figure 4A). In contrast, SU6656 did not alter Smad2 phosphorylation by TGFβ even at high doses (Figure 4A). These data indicate that Src family kinase functions downstream of TβRII in transducing Smad-independent TGFβ signaling to activate ATF2. Since rapid Src activation by TGFβ has been found to effectively activate Small GTPase RhoA in other cell systems [21], we then examined whether in VSMCs RhoA was activated downstream of Src family kinase. Indeed, TGFβ enhanced RhoA activation and SU6656 inhibited TGFβ-induced RhoA activation (Figure 4B). Furthermore, transfection of VSMCs with control or RhoA siRNA revealed that RhoA knockdown attenuated TGFβ-induced ATF2 activation but not that of Smad2 (Figure 4C). In addition, siRNA-mediated silencing of RhoA not only decreased activation of the signaling molecules at the early time point (Figure 4C) but also reduced TGFβ-induced CRP2 protein expression after 24 h (Figure 4D). These results suggest that Src family kinase-RhoA signaling mediates TβRII-dependent ATF2 activation and CRP2 expression by TGFβ.

**Figure 5:**
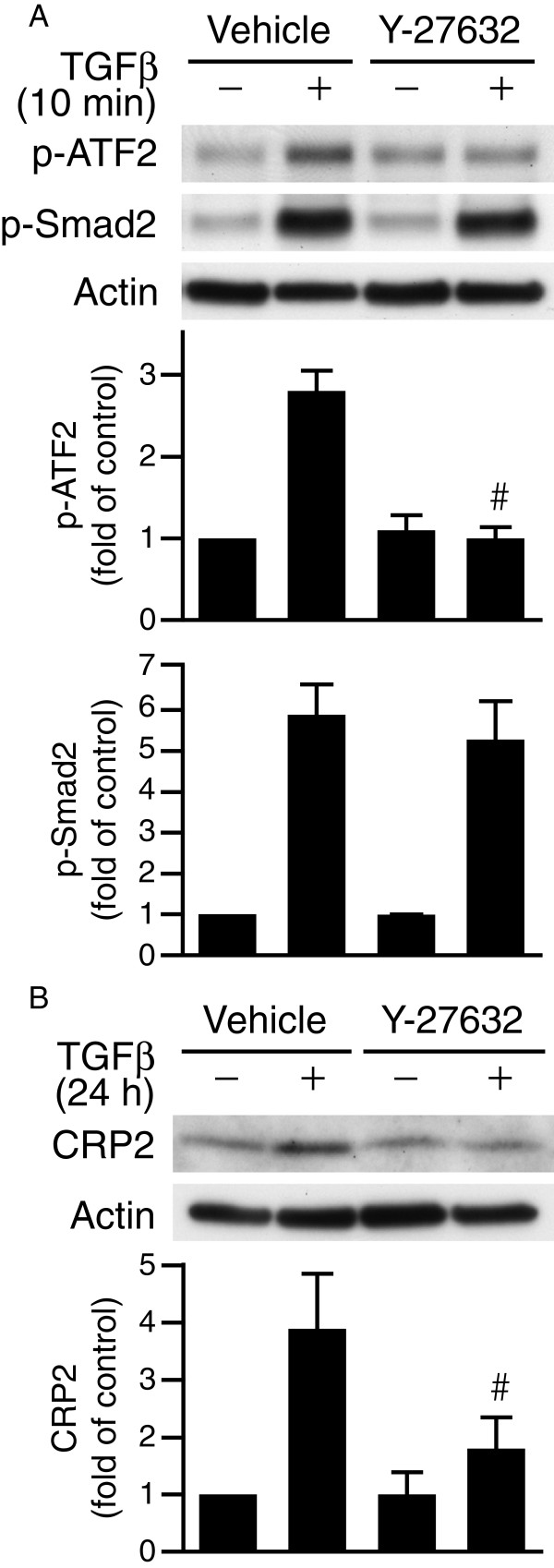
**ROCK mediates Smad-independent ATF2 activation by TGFβ. (A)** VSMCs were pretreated with vehicle or ROCK inhibitor Y-27632 (10 μM) for 30 min before stimulation with TGFβ for 10 min. Western blot analyses were then performed to examine phosphorylation of ATF2 and Smad2. ^#^*P* < 0.05 vs. TGFβ-stimulated vehicle group. **(B)** VSMCs were pretreated with vehicle or Y-27632 for 30 min before stimulation with or without TGFβ for 24 h. CRP2 expression was detected by Western blot analysis. ^#^*P* < 0.05 vs. TGFβ-stimulated vehicle group. Representative blots of at least three independent experiments are shown.

Since RhoA kinase (ROCK) is a major target of RhoA and that ROCK has been implicated in SMC differentiation by modulating TGFβ-Smad signaling [23], we examined whether ROCK functioned downstream of RhoA to regulate ATF2 activation. Interestingly, treatment of VSMCs with ROCK inhibitor Y-27632 abolished ATF2 phosphorylation but not that of Smad2 (Figure 5A). Supporting this notion, Y-27632 abrogated TGFβ-induced CRP2 protein expression after 24 h (Figure 5B).

### JNK activation is required for TGFβ-induced phosphorylation of ATF2

**Figure 6:**
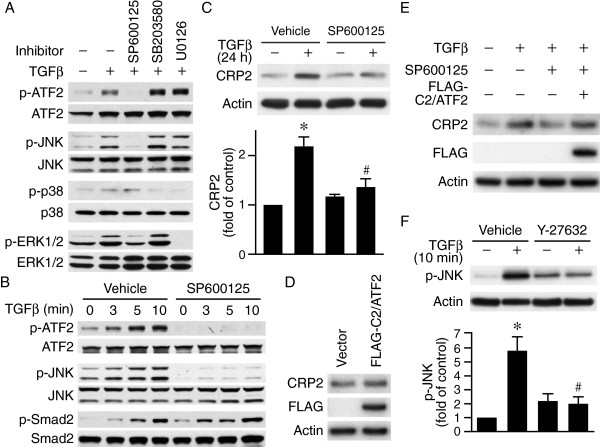
**JNK activation is required for TGFβ-induced ATF2 phosphorylation and CRP2 induction. (A)** JNK activation is required for TGFβ-induced phosphorylation of ATF2 but not Smad2/3. VSMCs were pretreated with vehicle, JNK inhibitor SP600125 (10 μM), p38 inhibitor SB203580 (10 μM), or ERK1/2 inhibitor U0126 (10 μM) for 30 min before stimulation with TGFβ (10 ng/ml). Phosphorylation of ATF2, JNK, p38, and ERK1/2 was determined 15 min after TGFβ stimulation. The membranes were subsequently probed with antibodies against total proteins for ATF2, JNK, p38, and ERK1/2 for loading control. **(B)** VSMCs were pretreated with vehicle or SP600125 for 30 min before stimulation with TGFβ. Cell lysates were harvested at the indicated time points and the phosphorylation of ATF2, JNK, and Smad2 examined. The membranes were subsequently probed with antibodies against total proteins for ATF2, JNK, and Smad2 for loading control. **(C)** JNK activity contributes to CRP2 induction. VSMCs were pretreated with vehicle or SP600125 for 30 min before stimulation with TGFβ for 24 h. Total proteins were then harvested for Western blot analysis to detect CRP2 expression. Values are mean ± S.E. of at least three experiments. *P < 0.05 vs. control (− TGFβ); ^#^*P* < 0.05 vs. TGFβ-stimulated vehicle group. **(D)** Constitutively active FLAG-C2/ATF2 increases CRP2 expression. VSMCs were electroporated with control vector or FLAG-C2/ATF2 and total proteins prepared 24 h later. Western blot analysis was performed to assess CRP2 levels. Overexpression of FLAG-C2/ATF2 was evaluated by probing Western blots with FLAG antibody. **(E)** Overexpression of FLAG-C2/ATF2 rescues SP600125-inhibited TGFβ induction of CRP2. VSMCs were electroporated with control vector or FLAG-C2/ATF2, serum-starved, pretreated with vehicle or JNK inhibitor SP600125 for 30 min before stimulation with TGFβ for 24 h. Western blot analysis was performed to assess CRP2 expression. FLAG-C2/ATF2 expression was evaluated with FLAG antibody. **(F)** VSMCs were pretreated with vehicle or ROCK inhibitor Y-27632 (10 μM) for 30 min before stimulation with or without TGFβ for 10 min. Phospho-JNK expression was detected by Western blot analysis. The membranes were subsequently probed with actin for loading control. *P < 0.05 vs. control (− TGFβ); ^#^*P* < 0.05 vs. TGFβ-stimulated vehicle group. Representative blots of at least three independent experiments are shown.

Because MAP kinase pathways have been shown to contribute to TGFβ signaling [24,25], we evaluated activation of MAP kinase pathway in ATF2 phosphorylation. TGFβ increased phosphorylation of JNK, p38, and ERK1/2 (Figure 6A). However, kinase inhibitor studies revealed that JNK inhibitor SP600125 but not p38 MAP kinase inhibitor SB203580 or ERK inhibitor U0126 abolished ATF2 phosphorylation (Figure 6A). Interestingly, SP600125 did not affect TGFβ-induced Smad2 phosphorylation (Figure 6B). To determine whether JNK inhibition ultimately affected CRP2 induction by TGFβ, we first pretreated VSMCs with vehicle or SP600125 for 30 min, stimulated with or without TGFβ for 24 h, then examined CRP2 expression levels. TGFβ induced CRP2 protein levels while SP600125 significantly reduced CRP2 levels (Figure 6C), suggesting JNK functions upstream of ATF2 to mediate CRP2 induction. Since unphosphorylated ATF2 is transcriptionally inactive, we overexpressed a constitutively active C2/ATF2 construct in VSMCs and examined CRP2 expression levels. Indeed, overexpression of C2/ATF2 increased CRP2 protein 1.5-fold (vs. vector control, *P* < 0.05) (Figure 6D). Furthermore, C2/ATF2 rescued the TGFβ induction of CRP2 that was inhibited by JNK inhibitor SP600125 (Figure 6E), demonstrating the importance of JNK-ATF2 pathway in TGFβ-mediated CRP2 induction. We next wanted to determine whether JNK functioned downstream of ROCK in the signaling pathway leading to ATF2 activation. Indeed, Y-27632 abrogated TGFβ-induced JNK activation (Figure 6F), linking ROCK-JNK-ATF2 signaling axis.

### Both SBE and CRE sites are functionally important for basal and TGFβ induction of the *Csrp2* promoter activity

**Figure 7:**
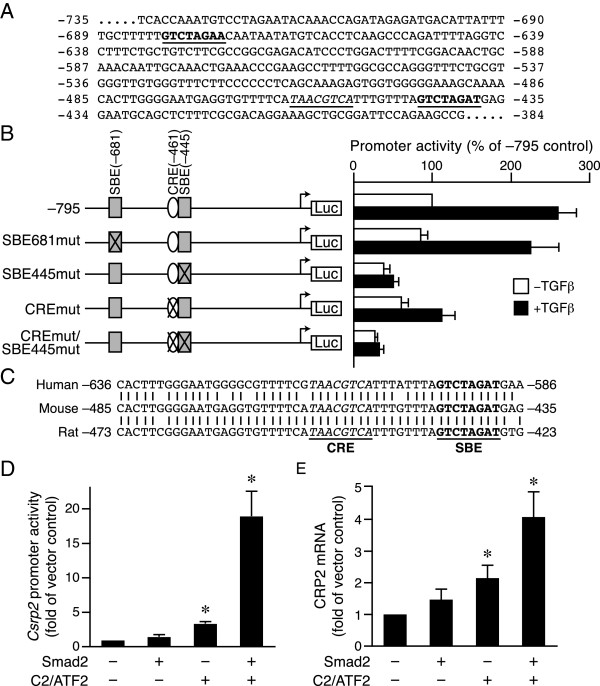
**SBE445 and CRE461 are functionally important for basal and TGFβ-induced *Csrp2 *promoter activity. (A)** Two putative SBE sites at bp −681 and −445 of the mouse *Csrp2* promoter (bold and underlined). The CRE site at −461 is also indicated (italic and underlined). **(B)** The putative SBE site at −445 and CRE at −461 are important for *Csrp2* promoter activity. The −795 *Csrp2* wild type and SBE and CRE mutant promoter constructs are schematically depicted in the left panel. VSMCs were transiently transfected with *Csrp2* luciferase reporter constructs containing −795, SBE681mut, SBE445mut, CREmut, or CREmut/SBE445mut in triplicate using FuGENE 6 transfection reagent. Two hours after transfection, cells were treated with or without TGFβ (10 ng/ml) for 24 h. Cells were then harvested for luciferase activity and protein assays. Luciferase activity is expressed relative to −795 without TGFβ treatment. Values are mean ± S.E. of at least three experiments. **(C)** Conservation of the CRE and SBE sites among species. Sequence alignment of the corresponding regions of human and rat promoter sequences to the mouse promoter. The CRE site is in italic and SBE site in bold type. **(D)** VSMCs were transiently cotransfected with –795*Csrp2*-luc reporter with empty vector or expression plasmids Smad2, C2/ATF2, or both. Cells were then harvested 24 h later for luciferase activity and protein assays. Luciferase activity is expressed relative to −795 with empty expression vector. Values are mean ± S.E. of at least three experiments. *P < 0.05 vs. empty expression vector. **(E)** VSMCs were electroporated with control vector or expression plasmids Smad2, C2/ATF2, or both. Total RNA was prepared 12 h later for real-time PCR analysis to assess CRP2 mRNA expression and β-actin was used as an internal control for normalization. Quantification was performed by the comparative C_T_ method. CRP2 mRNA is expressed relative to empty expression vector. Values are mean ± S.E. of at least three experiments. *P < 0.05 vs. empty expression vector.

It is evident from our studies that Smad2/3 and ATF2 participate in the TGFβ induction of CRP2. As ATF2 activates *Csrp2* transcription via CRE site [13], we set out to identify elements that are responsible for Smad2/3-mediated induction. Examination of the sequences within the mouse −795 bp *Csrp2* promoter (which is responsive to TGFβ) revealed that in addition to the previously identified CRE site at bp −461 (Figure 7A, italic and underlined) there are two potential Smad binding elements (SBE), located at bp −681 and −445 (Figure 7A, bold and underlined), each with a base divergent from the consensus SBE (5′-GTCTAGAC-3′). To determine the potential function of these two putative SBE sites, we generated SBE mutant luciferase constructs, −795SBE681mut and –795SBE445mut, each with 3 bases mutated within the putative SBE sites (Figure 7B, left). We then transiently transfected VSMCs with parental wild-type luciferase plasmid –795*Csrp2*-luc and mutant constructs. Mutation of SBE681 did not affect either basal or TGFβ induction of *Csrp2* promoter activity (Figure 7B). Similar to that of CRE mutation, SBE445 mutation not only decreased basal promoter activity but also reduced TGFβ responsiveness (Figure 7B). Double mutation of CRE and SBE445 further reduced promoter response (Figure 7B). These results indicate that both the SBE at bp −445 and CRE at −461 are functionally important in regulating *Csrp2* transcription. Further supporting this notion, these two sites are completely conserved across species among human, mouse, and rat (Figure 7C).

To further demonstrate the critical roles of Smad2/3 and ATF2 in *Csrp2* transcriptional regulation, we cotransfected luciferase plasmid –795*Csrp2*-luc with expression plasmids Smad2, C2/ATF2, or both in VSMCs and examined promoter activity. Smad2 slightly increased *Csrp2* promoter activity (1.5-fold, Figure 7D) although it did not reach a statistical significance, likely because Smad 2 might be inactive without TGFβ stimulation. Constitutively active C2/ATF2 significantly increased *Csrp2* promoter activity to 3.4-fold (Figure 7D). Interestingly, Smad2 and C2/ATF2 together enhanced *Csrp2* promoter activity to 19-fold (Figure 7D), indicating a synergistic effect of these two factors. Next, we performed real-time PCR to examine the effects of Smad2 and C2/ATF2 on CRP2 mRNA levels. Similar to promoter activity, Smad2 slightly increased while C2/ATF2 increased CRP2 mRNA levels to 2-fold (Figure 7E). In addition, Smad2 and C2/ATF2 synergistically induced CRP2 mRNA levels (Figure 7E).

## Discussion

CRP2 plays a critical role in attenuating the development of arteriosclerosis [3,4]. Upregulating CRP2 in the injured artery may protect against neointima formation. The goal of this study was to identify mechanisms and signaling pathways that sustain or upregulate CRP2 expression to decrease vascular disease. We show that activation of both Smad2/3 and ATF2 are essential for enhancing CRP2 expression. Unlike the TβRI-dependent Smad pathway, Src family kinase-RhoA-ROCK-JNK signaling axis mediates TβRI-independent ATF2 activation. The TGFβ induction of CRP2 is mediated through the CRE and SBE promoter elements. Our results demonstrate that two signaling axes downstream of TGFβ converge on *Csrp2* promoter to cooperatively control CRP2 induction in VSMCs.

Smad proteins function as intracellular TGFβ signaling effectors. Following activation and translocation into the nucleus, the heterotrimeric Smad complex recognizes and binds to SBE site to activate target gene expression [26]. Consistent with this canonical signaling pathway, we found in VSMCs that TGFβ activated Smad2/3 to induce CRP2 expression via a SBE site at bp −445 of the *Csrp2* promoter (Figures 1 and 7). This Smad2/3 activation is dependent on TβRI and its kinase activity, as siTβRI or SB431542 inhibited TGFβ-induced Smad2/3 phosphorylation (Figures 1 and 3). TGFβ also activated ATF2 to induce CRP2 expression ( [13] and Figure 1). The fact that ATF2 phosphorylation was not affected either by inhibiting TβRI kinase activity or knocking down TβRI expression (Figures 1B and 3A) indicated that TβRI was dispensable for ATF2 activation. By contrast, TβRII was required for both Smad2 and ATF2 activation (Figure 3C). Intriguingly, although it was expected that overexpression of DN-TβRII inhibited Smad2 phosphorylation (because it lacks cytoplasmic C-terminal kinase domain and is unable to activate TβRI) DN-TβRII failed to inhibit ATF2 phosphorylation (Figure 3D). Taken together, these results suggest that in VSMCs TβRII alone is able to mediate TGFβ signaling to ATF2 and the cytoplasmic domain of TβRII is not required. This TβRI-independent signaling to ATF2 is similar to the findings in dermal cells that TβRII directly activates ERK1/2 without the participation of TβRI [27] and supports the notion that TGFβ receptors can activate non-Smad proteins and allow Smad-independent TGFβ responses [28].

We found JNK functioned upstream of ATF2 in the TGFβ induction of CRP2 (Figure 6); however, JNK inhibition did not affect Smad2/3 activation (Figure 6B). These results are in contrast to the findings in Mv1Lu epithelial cells and T98G glioblastoma cells [29,30]. In epithelial cells, while TGFβ activates both Smad3 and JNK there is an interdependent Smad and JNK signaling because activated JNK in turn phosphorylates Smad3 [29]. In glioblastoma cells [30], TGFβ activates p38 MAPK and ATF2 but does not induce JNK phosphorylation; furthermore, inhibition of p38 MAPK decreases TGFβ-induced phosphorylation of ATF2 and Smad2. Those findings indicate an interaction between Smad and p38 MAPK pathways downstream of TGFβ [30] that are in contrast to our results that p38 MAPK and ERK1/2 are not involved in ATF2 activation (Figure 6A). Our current findings and these previous reports suggest that TGFβ signaling pathways are likely to be cell type specific.

We demonstrated that both CRE and SBE sites contribute to CRP2 upregulation by TGFβ (Figure 7). Importantly, these two sites are completely conserved across species among human, mouse, and rat (Figure 7C), suggesting the critical importance of the CRE and SBE in the regulation of *Csrp2* transcription. The importance of CRE in mediating TGFβ target gene induction was shown in a rat intestinal epithelial cell line 4–1 that transcription factors CREB-1, ATF2, c-Jun, and Smad3 all bind to the CRE site to activate transcription [31]. Interestingly, ATF2, via its basic leucine zipper region, was shown to bind to the MH1 domain of the Smad proteins [32]. TGFβ stimulation further enhances the association of ATF2 and Smad3/4 and increases the CRE-containing reporter activity in HepG2 cells [32]. Since blocking either the ATF2-CRE or TβRI-Smad2/3 axis significantly reduced CRP2 expression (Figures 1 and 7) and given the proximity of CRE and SBE sites (8 bp apart), it is possible that Smad heterotrimer and ATF2 might form a higher order complex to cooperatively, rather than independently, regulate CRP2 expression. This regulation via two *cis* elements is similar to that of several SMC marker genes [33]. For example, the TGFβ induction of SM α-actin and SM22α expression is coordinately regulated by a TGFβ control element (TCE) and nearby CArG (CC(A/T)_6_GG) elements in the 5′ promoter [33,34]. However, the regulatory mechanism of CRP2 expression by TGFβ differ from these SMC marker genes in that CRP2 induction is mediated through the CRE and SBE elements whereas TCE and CArG elements mediate the TGFβ induction of SM α-actin and SM22α expression.

## Conclusion

**Figure 8:**
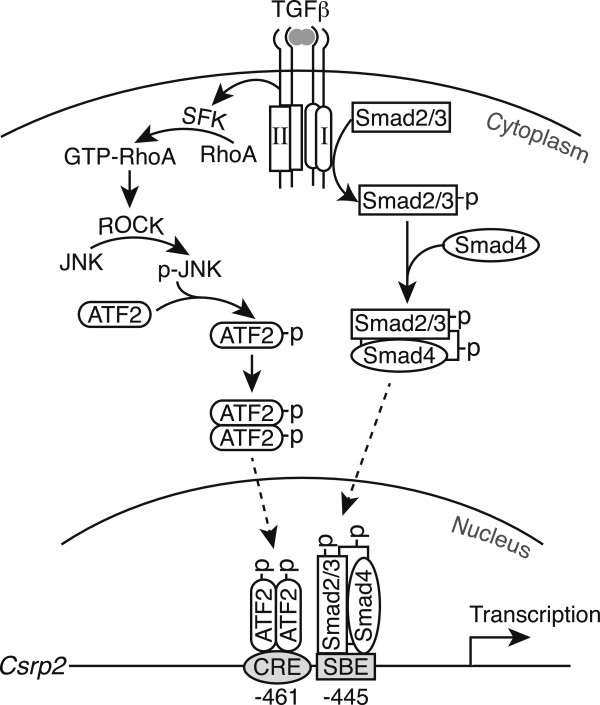
**Schematic model of TGFβ signaling pathways for CRP2 induction in VSMCs.** Upon TGFβ stimulation, two signaling axes are activated: the canonical TβRI-dependent Smad2/3 pathway and the non-canonical ATF2 pathway. In the canonical pathway, activated Smad2/3 form a complex with Smad4, translocates into the nucleus, and binds to SBE site at −445 of the *Csrp2* promoter. In the non-canonical pathway, following TβRII binding of TGFβ, TβRII (without the requirement of TβRII’s cytoplasmic kinase domain) activates Src family kinase (SFK), which in turn activates RhoA. Enhanced RhoA activation leads to ROCK activation and increased JNK phosphorylation, resulting in enhanced ATF2 phosphorylation. Activated ATF2 dimer binds to CRE site at −461. The two signaling pathways (ATF2 and Smad) converge on the CRE and SBE sites of the *Csrp2* promoter to cooperatively control CRP2 induction in VSMCs, which represents a previously unrecognized mechanism of VSMC gene induction by TGFβ.

Based on our findings, we propose that two signaling pathways downstream of TGFβ converge on *Csrp2* promoter to cooperatively induce *Csrp2* gene transcription and protein expression (Figures 1, 7, and 8). In the canonical pathway, upon TGFβ binding, TβRII trans-phosphorylates TβRI to activate its kinase function, leading to Smad2/3 phosphorylation. Activated Smad2/3 then cooperate with Smad4 to form a complex, translocates into the nucleus and bind to SBE of the *Csrp2* promoter. In the non-canonical signaling pathway that does not require TβRI, TGFβ binds to TβRII and activates Src family kinase and RhoA signaling. Activated ROCK in turn phosphorylates JNK, resulting in ATF2 activation. Activated ATF2 dimer then binds to the CRE site 8 bp upstream of SBE on the *Csrp2* promoter. The ATF2 on the CRE site and Smad proteins on the SBE site might associate to form a higher order complex to cooperatively enhance CRP2 expression (Figure 8) in VSMCs, which represents a previously unrecognized mechanism of VSMC gene induction by TGFβ.

## Methods

### Luciferase reporter and expression constructs

The –795*Csrp2*-luc luciferase reporter plasmid was described previously [35] and used as a template to generate mutant constructs. Site-directed mutagenesis was performed using Pfu polymerase (Stratagene) to mutate the putative SBE sites at −681 from GTCTAGAA to *C*T*A*T*C*GAA and at −445 from GTCTAGAT to *C*T*A*T*C*GAT (mutated bases are italicized) to generate SBE681mut and SBE445mut constructs, respectively. The CREmut/SBE445mut construct with double mutations at –461CRE and –445SBE was generated using –795CREmut*Csrp2*-luc [13] as a template to mutate putative –445SBE site as above. All constructs were confirmed by DNA sequencing. Dominant-negative mutant of type II TGFβ receptor pCMV5 HA-TβRII(∆Cyt) that lacks C-terminal kinase domain and is unable to activate TβRI [20] was obtained from Addgene (plasmid 14051). The expression plasmid pCMV5-Smad2 has previously been described [36]. The constitutively active mutant construct FLAG-C2/ATF2 [37] that contains a FLAG tag at the N-terminus, followed by the constitutively active activation domain of CREB2 and the DNA binding and dimerization domains of ATF2 was generously provided by G. Thiel (University of the Saarland Medical Center, Homburg, Germany).

### VSMC primary culture and transient transfection assays

Primary VSMCs were isolated from mouse aortas and cultured in Dulbecco’s modified Eagle’s medium (DMEM) as described [38]. Cells of passages 5–8 were used for experiments. The day before transfection, approximately 160,000 VSMCs were plated onto each well of 6-well plates. Cells were then transfected with various luciferase reporter constructs (1 μg/well) in triplicates by using FuGENE 6 or X-tremeGENE 9 according to the manufacturer’s instructions (Roche). Two hours following transfection, cells were treated with or without TGFβ (PeproTech, 10 ng/ml). Luciferase activities were measured 24 h later using Luciferase Assay System (Promega) and normalized to total protein content. To evaluate the effect of Smad2 and ATF2 on promoter activity, cells were cotransfected with –795*Csrp2*-luc (0.33 μg/well) and 0.67 μg/well of expression plasmid (empty vector, CMV5-Smad2, FLAG-C2/ATF2, or Smad2 + C2/ATF2) and luciferase activity measured 24 h later.

For overexpression experiments, VSMCs were electroporated with vector control or expression plasmid Smad2, C2/ATF2, or Smad2 + C2/ATF2 (8 μg DNA/1 × 10^6^ cells) with Gene Pulser Xcell™ Electroporation System (Bio-Rad) using 400 V, 10 ms, square wave parameters. Following recovery, cells were serum starved (0.5% FBS in DMEM) and total RNA or protein isolated at indicated time points for real time-PCR or Western blot analysis. For dominant-negative TβRII experiments, VSMCs were electroporated with pCMV5 vector or pCMV5 HA-TβRII(∆Cyt), serum starved, treated with or without TGFβ, and then total proteins isolated for Western blot analysis at different time points.

### Western blot analysis

To evaluate the effects of TGFβ on protein expressions and downstream signaling, VSMCs were plated and incubated overnight. Following serum-starvation (0.5% FBS in DMEM) for 24 h, cells were stimulated with or without TGFβ. Total proteins were prepared at the indicated time points using extraction buffer containing protease inhibitor Complete™ (Roche) and Halt™ Phosphatase Inhibitor Cocktail (Thermo Scientific) for Western blot analysis as described [13]. To inhibit various kinase activities, VSMCs were treated with inhibitors 30 min prior to stimulation with or without TGFβ. SB431542 (Calbiochem) was used for inhibiting TβRI kinase activity, Wortmannin and LY294002 (Calbiochem) for PI3K, SP600125 (Calbiochem) for JNK, SB203580 (Calbiochem) for p38, U0126 (Calbiochem) for ERK1/2, Y-27632 (Calbiochem) for ROCK, and SU6656 (Sigma-Aldrich) for inhibiting Src family kinase activity.

To assess phosphorylated and total protein of signaling molecules, membranes were incubated with antibodies against respective phospho- or total protein. The following antibodies were purchased from Cell Signaling Technology: phospho-ATF2 (Thr^71^, #9221), phospho-Smad2 (Ser^465/467^, #3108), phospho-Smad3 (Ser^423/425^, #9520), Smad2/3 (#3102), phospho-JNK Thr^183^/Tyr^185^ (#9251), JNK (#9252), phospho-p38 Thr^180^/Tyr^182^ (#9211), p38 (#9212), phospho-ERK1/2 Thr^202^/Tyr^204^ (#9101), ERK1/2 (#9102), and TβRIII (#2519). ATF2(C19) antibody (Santa Cruz Biotechnology) was used to detect total ATF2. CRP2-(91–98) antiserum [13] was used for CRP2 protein detection. TAK1 and TRAF6 antibodies (Santa Cruz Biotechnology) were used to detect TAK1 and TRAF6 expression. To detect dominant-negative TβRII (HA-TβRII(∆Cyt)) expression, an anti-HA tag antibody (114-2C-7) (Millipore, #05-902R) was used. Antibodies TβRI (V-22) (Santa Cruz Biotechnology), TβRII (L-21) (Santa Cruz Biotechnology), and TβRIII (Cell Signaling Technology, #2519) were used to detect endogenous TβRI, TβRII, and TβRIII expression, respectively. A mouse monoclonal anti-FLAG antibody (Sigma, #F9291) was used to detect FLAG-C2/ATF2 expression. To verify equivalent loading, membranes were subsequently incubated with an actin antibody (Millipore, MAB1501).

### siRNA knockdown

To suppress mRNA levels of Smad2/3, ATF2, TAK1, TRAF6, RhoA, TβRI, TβRII, and TβRIII, we performed knockdown experiments with small interfering RNA (siRNA). The ON-TARGET plus SMART pool siRNAs and negative control siRNA were obtained from Dharmacon. VSMCs (2.8 x 10^5^ cells) were plated on 60-mm dish and after overnight incubation, cells were transfected with 20 nM of negative control or target siRNA in Opti-MEM® I Reduced Serum Medium using Lipofectamine RNAiMAX as described by the manufacturer’s protocol (Life Technologies).

### Assessment of RhoA activation

RhoA activation was assessed using an Active Rho Pull-Down and Detection Kit (Thermo Scientific) by immunoprecipitating GTP-bound RhoA with GST fusion protein of Rhotekin RhoA binding domain (GST-Rhotekin-RBD). Briefly, VSMCs plated in 150-mm dish were washed with ice-cold PBS and lysed in buffer containing 25 mM Tris pH 7.2, 150 mM NaCl, 5 mM MgCl_2_, 1% NP-40, and 5% glycerol. Cell lysates (500 μg) were incubated with 400 μg of GST-Rhotekin-RBD and glutathione resin at 4 °C for 1 h. Following washing, bound Rho was eluted by SDS sample buffer. The eluted samples and the total cell lysates were then subjected to Western blot analysis with RhoA antibody (26C4) (SantaCruz Biotechnology, sc-418) to detect active and total RhoA, respectively.

### Quantitative real time-PCR

Total RNA was isolated using an RNeasy Mini kit (QIAGEN) according to the manufacturer’s instructions. One μg RNA was first reverse transcribed to cDNA with random primers using SuperScript III reverse transcriptase (Invitrogen). Quantitative real time-PCR was performed with the transcribed cDNA and SYBR® FAST Universal 2X qPCR Master Mix (KAPA) in triplicates using the 7500 real time PCR system (Applied Biosystems) to detect CRP2 mRNA expression. The primers used for CRP2 are: forward, 5′-CTGACTGAGAAAGAAGGCGAAATC-3′ and reverse, 5′-TGCTGGCTGTTTCACAGTAGTGA-3′. β-actin was used as an internal control for normalization with the following primers: forward, 5′-GAGAGGTATCCTGACCCTGAAG-3′; reverse, 5′-TGATCTGGGTCATCTTTTCACGG-3′. Quantification was performed by the comparative C_T_ method.

### Statistical analysis

Data are presented as mean ± S.E. of at least three independent experiments. All results were analyzed statistically by Student’s *t*-test. *P* values <0.05 are considered statistically significant.

## Abbreviations

CArG element: CC(A/T)_6_GG; CRE: Cyclic AMP response element; CRP2: Cysteine-rich protein 2; Csrp2: Mouse CRP2 gene symbol; ROCK: RhoA kinase; SBE: Smad binding element; siRNA: Small interfering RNA; SM: Smooth muscle; TβR: TGFβ receptor; TCE: TGFβ control element; VSMCs: Vascular smooth muscle cells.

## Competing interests

The authors declare no competing interests.

## Authors’ contributions

MLW, CHC, YTL, YJJ, and YCH performed experiments, MLW, LTY, LC, MDL, and SFY analyzed and interpreted the data, MLW, MDL, and SFY prepared the figures. MLW, MDL, and SFY designed the research and wrote the manuscript. All authors had a final approval of the manuscript.
